# Prevalence and distribution of dermatophytosis lesions on cattle in Plateau State, Nigeria

**DOI:** 10.14202/vetworld.2019.1484-1490

**Published:** 2019-09

**Authors:** J. S. Dalis, H. M. Kazeem, J. K. P. Kwaga, C. N. Kwanashie

**Affiliations:** 1 Department of Veterinary Microbiology, Faculty of Veterinary Medicine, Ahmadu Bello University, Zaria, Nigeria; 2 Department of Veterinary Public Health and Preventive Medicine, Faculty of Veterinary Medicine, Ahmadu Bello University, Zaria, Nigeria

**Keywords:** cattle, dermatophytes, identification, isolation, Nigeria, Plateau State

## Abstract

**Background and Aim:**

Dermatophytosis is an infection of the superficial, keratinized structures of the skin, nails, and hair of man and animals caused by a group of fungi called dermatophytes in the genera *Trichophyton*, *Microsporum*, and *Epidermophyton*. The prevalence of dermatophytosis among cattle in Nigeria and Plateau State, in particular, is yet to be fully determined. This study aimed to determine the prevalence and the distribution of dermatophytosis lesions on cattle in Plateau State, Nigeria.

**Materials and Methods:**

Four hundred and thirty-seven cattle showing visible skin lesions suggestive of dermatophytosis were drawn from nine local government areas (three each) from the three senatorial districts of Plateau State, Nigeria. Skin scrapings were aseptically collected using a cross-sectional study, in which sampling units were selected using purposive sampling method. Samples were processed for both direct microscopic examination and isolation of dermatophytes in culture. The isolates were stained with lactophenol cotton blue and identified microscopically based on the size, shape, and arrangement of macro- and micro-conidia. The dermatophytes were further identified by determining the sequences of the internal transcribed spacer regions of their ribosomal DNA. Data were analyzed and presented as percentages, bar graph, and Chi-square test of association. p≤0.05 was considered statistically significant.

**Results:**

The overall prevalence rate of bovine dermatophytosis in Plateau State was found to be 11.0%. *Trichophyton verrucosum* was more frequently isolated (54.2%) than *Trichophyton mentagrophytes* (45.8%). Age, breed, management practice, and season were significantly associated with the occurrence of the disease (p<0.05).

**Conclusion:**

Dermatophytosis among cattle may be of public health significance in Plateau State, Nigeria. This is the first report on the prevalence and distribution of dermatophytosis lesions on cattle from Plateau State, Nigeria.

## Introduction

Dermatophytosis also known as tinea or ringworm, is an infectious, highly contagious skin disease that affects animals as well as humans. The disease is caused by a group of keratinophilic filamentous fungi called dermatophytes. The dermatophytes are classified into the genera *Trichophyton*, *Microsporum*, and *Epidermophyton* based on conidia morphology and accessory organs [1]. However, De-Hoog *et al*. [2] in a multilocus phylogenetic study of the family *Arthrodermataceae* found the genus *Trichophyton* to be polyphyletic and proposed nine genera for the dermatophytes including *Trichophyton*, *Epidermophyton*, *Nannizzia*, *Paraphyton*, *Lophophyton*, *Microsporum*, *Arthroderma*, *Ctenomyces*, and *Guarromyces*.

Dermatophytosis is an important public health problem worldwide, impacting millions of individuals annually [3]. It is the most frequently encountered dermatologic problem in veterinary practice affecting a wide range of domestic and wild animals [4]. Ambilo and Melaku [5] in a study of the major skin diseases affecting cattle in Ethiopia found that dermatophytosis was the most common skin disease affecting the bovine species. In animal husbandry, losses may be incurred due to the high cost of treatment, weight loss, decrease in milk production, and the poor quality of rawhide materials in view of hides and skins being affected and destroyed by dermatophytes. Consequently, a national vaccination program against the disease was advocated by the Swedish hide industry [6].

In Nigeria, reports on the prevalence of dermatophytosis in domestic animals and cattle, in particular, are scanty [7]. Earlier studies on dermatophytoses of domesticated animals in different parts of the country in Ibadan, Oyo State [8], in Zaria, Kaduna State [9], in Oyo State [10] and a more recent report from Nsuka, Enugu State [11], did not cover cattle. In the study conducted in Enugu, Anambra, Ebonyi, Abia, Imo, Kogi and Delta States of Nigeria [12], only 55 out of a total of 538 animals in that study were cattle. Apart from the gross underrepresentation of the Nigerian cattle population in the previous studies, only two locations, Zaria, in Kaduna State [9] and in Kogi State [12], were from the northern part of the country, which has over 85% of the total cattle population in Nigeria [13].

It is quite obvious, therefore, that the prevalence of bovine dermatophytosis in Nigeria and Plateau State, in particular, is yet to be fully determined. This study investigates and documents the prevalence rates and the distribution of dermatophytosis lesions on cattle in Plateau State, Nigeria.

## Materials and Methods

### Ethical approval

This type of research (removal of keratinized dead scales which does not inflict pain to an animal and in fact, constitutes a form of treatment for the disease) does not require ethical clearance in our laws and regulations.

### Study area

The study was conducted on cattle in Plateau State, located in North Central Nigeria. Its coordinates are 9^o^10 ‘0”N and 9^o^45’0”E in (Degrees, Min, S). Sampling locations: Bassa, Jos North, and Jos South Local Government Areas (LGAs) in Plateau North senatorial district; Mangu, Pankshin, and Kanam LGAs in Plateau Central senatorial district; and Wase, Mikang, and Shendam LGAs in Plateau South senatorial district were selected by simple random sampling technique [14] by balloting. Samples were collected based on a cross-sectional study, in which sampling units were selected using the purposive sampling method [15].

A total of 4753 cattle were physically examined for skin lesions consistent with dermatophytosis. Four hundred and thirty-seven skin scrapings were collected by first, cleaning the lesion site using cotton wool soaked in 70% alcohol to remove surface adhering organisms, and the edges of the lesions were scraped using sterile scalpel blade into the clean envelope. Age of animal (young < 1 year and adult > 1 year), sex, breed, and the anatomical location of lesions where samples were collected on each animal were recorded. Samples were labeled and transported to the Mycology Laboratory, Department of Veterinary Microbiology, Ahmadu Bello University, Zaria, and stored at room temperature until analyzed.

### Laboratory examination

Each specimen was divided into two parts. One part was used for direct microscopic examination and the other portions for the isolation of etiologic agent in culture. The direct examination was performed as described by Bhatia and Sharma [16]. Briefly, a portion of each specimen was placed in a drop of 20% potassium hydroxide (KOH) on a clean grease-free glass slide and covered with a coverslip. The preparation was gently heated over the flame from a Bunsen burner to facilitate digestion. The slides were examined using 10× and 40× objectives of a light microscope (Nikon, ECLIPSE-E100, 824592, China) for fungal elements. The presence of hyaline septate hyphae in skin scales or spores inside or outside the hair shaft was considered positive for dermatophytes.

Isolation of dermatophytes was conducted using the method described by Robert and Pihet [17]. Each of the skin samples was also inoculated onto a Petri dish containing Sabouraud’s dextrose agar incorporated with cycloheximide at 0.5 mg/ml and chloramphenicol at 16 μg/ml. The specimens were placed directly and pressed into the medium with an inoculating loop to ensure adequate contact between specimen and medium. The plates were sealed with masking tape, incubated at 37°C, and observed for fungal growth every 3 days for a period of 21-30 days.

### Identification of fungal isolates

Information on colony features such as pigmentation (surface and reverse sides), topography (flat, heaped, regularly, or irregularly folded), texture (yeast-like, glabrous, powdery, granular, velvety, or cottony), and rate of growth (slow or rapid) were noted. Microscopic identification of dermatophytes was performed as described by Robert and Pihet [17]. Briefly, a portion of mycelium was transferred into a drop of lactophenol cotton blue on a clean grease-free glass slide and teased with a 22 gauge nichrome needle to separate the filaments. A coverslip was placed on the preparation and examined with 10× and 40× objectives of a light microscope using a much-reduced light for identification. Dermatophytes were identified based on variations in size and shape of macroconidia and microconidia, chlamydoconidia, spiral hyphae, nodular organs, and pectinate branches [17]. The isolates were further identified by determining the sequences of the internal transcribed spacer (ITS) regions of their ribosomal DNA [2]. The DNA was extracted using ZR Fungal/Bacterial DNA Kit^™^ Catalog No. D 6005 (Zymo Research Corporation) following the manufacturer’s instructions. The PCR amplification of the ITS regions of ribosomal DNA (rDNA) was carried out using the primers ITS-1 (5’-TCCGTAGGTGAACCTGCGG-3’) and ITS-4 (5’-TCCTCCGCTTATTGATATGC-3’) as the forward and reverse primers, respectively [18]. Amplification products were separated by electrophoresis in 1.5% agarose gels incorporated with ethidium bromide and documented using gel documentation system (BIORAD Laboratories). The amplified DNA was purified using Wizard PCR Preps DNA Purification System (Promega) and sequenced with the primers ITS-1 (5’-TCCGTAGGTGAACCTGCGG-3’) and ITS-4 (5’-TCCTCCGCTTATTGATATGC-3’) using the dye terminator method (Applied Biosystems 377 automatic sequencer). The partial sequences of the ITS1, 5.8S, and partial ITS2 regions of the ribosomal DNA were compared at nucleotide positions 76-587 with the sequences of the ITS1, 5.8S, and ITS2 of *Trichophyton verrucosum* and *Trichophyton mentagrophytes* available in the Gene Bank for identification.

## Statistical analysis

Data were analyzed using SPSS version 21(IBM, USA). p≤0.05 was considered statistically significant.

## Results

### Clinical signs

**Figure-1:**
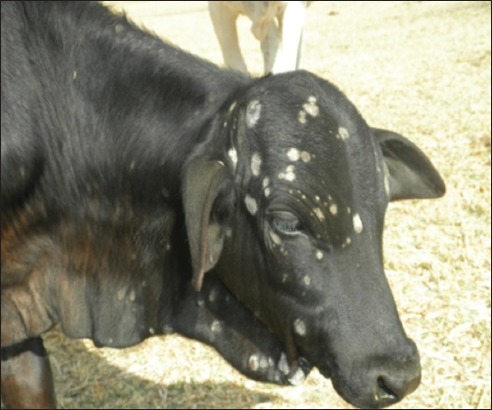
An 8-month-old Friesian-Bunaji crossbred calf, showing ringworm lesions. Circumscribed, grayish-white, thick, crusty, and raised lesions on the head region (arrow).

Skin lesions were discrete, alopecic, circular, circumscribed, crusty, and grayish-white, raised above the skin. Some animals had very few lesions on the head region, especially around the eyes while in other cases, many lesions involving the head, face, and dewlap were observed (Figure-1).

### Laboratory examination

Of 437 skin samples processed, 57 (13.0%) were positive for dermatophytes by direct examination, while 48 (11.0%) were positive by culture. All the samples that were negative through direct examination were also culture negative. Direct examination of samples revealed non-pigmented, septate hyphae in skin scales while ectothrix spores were observed on hair. Of the 48 dermatophytes isolated in culture, 22 (45.8%) and 26 (54.2%) were identified as *T. mentagrophytes* and *T*. *verrucosum*, respectively. Colonies of *T. mentagrophytes* were white, flat, and granular with yellow reverse color. Microscopically, many globose microconidia arranged in grape-like clusters were observed with elongated, pencil-shaped, thin, and smooth-walled macroconidia. Colonies of *T. verrucosum* were generally slow-growing, button or disk-shaped, white, glabrous, raised center, and flat periphery with some submerged growth and yellow reverse pigment. Microscopically, broad, irregular hyphae with abundant chlamydoconidia in chains (chains of pearls) were observed.

Comparing the sequences of the ITS regions of the ribosomal DNA of the isolates with the sequences of the ITS regions of the rDNA of dermatophytes available in the Gene Bank, 22 of the isolates were identified as *T. mentagrophytes*, whereas 26 were identified as *T. verrucosum* in complete agreement with the culture-based technique.

### Prevalence rates of bovine dermatophytosis based on sampling locations in Plateau State

Of the 48 dermatophytes isolated in culture, 28 (58%) including 15 of *T. verrucosum* and 13 of *T. mentagrophytes* were isolated from cattle in Plateau North Senatorial District, while 9 isolates (19%) including four of *T. verrucosum* and five of *T. mentagrophytes*, whereas11 isolates (23%) including seven of *T. verrucosum* and four of *T. mentagrophytes* were isolated from cattle in Plateau Central and Plateau South senatorial districts, respectively. The prevalence rate of bovine dermatophytosis was significantly higher (p<0.05) in Plateau North senatorial district when compared with Plateau Central senatorial and Plateau South senatorial districts. There was no significant difference in occurrence of the disease among cattle in Plateau Central and those in Plateau South senatorial districts (p>0.05) (Table-1).

**Table 1:** Prevalence rates of bovine dermatophytosis based on senatorial district in Plateau State, Nigeria.

Senatorial district	Number of samples tested	Number of dermatophytes isolated (%)	Species of dermatophytes isolated	

			*Trichophyton verrucosum* (%)	*Trichophyton* *mentagrophytes* (%)
Plateau North	167	28 (16.8)	15 (53.6)	13 (46.4)
Plateau Central	129	9 (7.0)	4 (44.4)	5 (55.6)
Plateau South	141	11 (7.8)	7 (63.6)	4 (36.4)
Total	437	48 (11.0)	26 (54.2)	22 (45.8)

χ^2^=10.37.*p=*0.006

### Prevalence rates of bovine dermatophytosis based on age, sex, breed, management system, and season in Plateau State

Of the 437 samples analyzed, 253 were obtained from adult cattle (> 1 year) while 184 were from young animals (≤ 1 year). Eleven (4.3%) adult and 37 (20.1%) young animals tested positive for dermatophytes. The prevalence rate of dermatophytosis was significantly higher (p<0.05) in young than adult cattle. Of the 275 male and 162 female animals tested, 27 (9.8%) males and 21 (13.0) females were positive for dermatophytes. There was no significant difference (p>0.05) in occurrence of the disease between male and female animals.

Of the 251 local Bunaji breed and 186 Friesian-Bunaji crossbred tested, 9 (3.6%) Bunaji breed and 39 (21.0%) Friesian-Bunaji crossbred were positive for dermatophytes. The prevalence of dermatophytosis was significantly higher (p<0.05%) in the Friesian-Bunaji crossbred than the local Bunaji breed. Of the 135 samples obtained from confined and 302 from unconfined cattle analyzed, 28 (20.7%) confined and 20 (6.6%) unconfined animals were positive for dermatophytes. The prevalence of dermatophytosis was significantly higher (p<0.05) in confined than unconfined animals. Thirty-nine (15.5%) of 251 cattle examined during the rainy season were positive for dermatophytes, whereas only 9 (4.8%) of the 186 animals examined during the dry season were positive for dermatophytes. The occurrence of the disease was significantly higher in the rainy than dry season (p<0.05) (Table-2).

**Table 2:** Prevalence of bovine dermatophytosis based on age, sex, breed, management system, and season in Plateau State, Nigeria.

Factor	Total	Positive	Prevalence rate (%)	χ^2^	OR (95% CI)	p-value
Age						
≤1 year	184	37	20.1	27.06	0.181 (0.089-0.365)	0.000
>1 year	253	11	4.3			
Sex						
Males	275	27	9.8	1.03	1.368 (0.746-2.509)	0.310
Females	162	21	13			
Breed						
Bunaji breed	251	9	3.6	33.01	7.134 (3.359-15.151)	0.000
Friesian-Bunaji cross	186	39	21.0			
Management system						
Confined	135	28	20.7	19.02	0.271 (0.146-0.502)	0.000
Not confined	302	20	6.6			
Season						
Rainy season	251	39	15.5	12.51	0.276 (0.130-0.586)	0.000
Dry season	186	9	4.8			

CI=Confidence interval, OR=Odds ratio

### Distribution of dermatophytosis lesions on the body of cattle based on age

Of a total of 37 young cattle that were positive for dermatophytes, ringworm lesions were seen most frequently on the head region, especially around the eyes in 70.3% (26/37), while 8.1% (3/37) of the lesions were found on the neck, 8.1% (3/37) on the back, 5.4% (2/37) on the dewlap, 5.4% (2/37) on the chest, and less frequently 2.7% (1/37) on the limbs. Similarly, of a total of 11 adult animals that were positive for dermatophytes, ringworm lesions occurred most frequently 27.3% (3/11) on the back, while 18.2% (2/11) of the lesions were found on the dewlap, 18.2% (2/11) on the neck, 18.2% (2/11) on the chest, and 9.1% (1/11) each on the head and limbs.

## Discussion

The overall prevalence rate of bovine dermatophytosis in Plateau State was found to be 11.0%. Of the species isolated, *T. verrucosum* was more frequently isolated (54.2%) than *T. mentagrophytes* (45.8%). Age, breed, management practice, and season were found to be significantly associated with the occurrence of the disease (p<0.05). Dermatophytosis lesions occurred more frequently on the head region (70.3%) than other parts of the body in young cattle, whereas in adult animals, the lesions were more common on the back (27.3%).

The prevalence rate of 11% bovine dermatophytosis in this study is similar to 12.6% reported by Nweze [12] who carried out a study on dermatophytosis of domestic animals involving 55 cows among other animals in seven Nigerian states. However, lower rates ranging from 4.5% to 8% have been reported in Perugia, Italy [19] while a much higher prevalence rate of 30.6% in Jordan [20]. These variations may be as a result of differences in geographical locations. The prevalence and causative agents of dermatophytosis may vary from one location to another depending on the population density, climatic and socioeconomic conditions, and natural reservoirs [16].

The isolation of *T. verrucosum* more frequently from ringworm lesions in this study suggests that it may be the most prevalent dermatophyte of cattle in the state. This finding is in agreement with the report of Shams-Ghahfarokhi *et al*. [21], Swai and Sanka [22], and Agnetti *et al*. [23] that *T. verrucosum* is the most common cause of ringworm in cattle. It is, however, different from the report of Ranganathan *et al*. [24] who found that *T. mentagrophytes* was the predominant cause of bovine dermatophytosis in India.

The main lesions of bovine dermatophytosis observed in this study were discrete alopecia, circular, circumscribed, thickly crusted, and grayish-white lesions raised above the skin which were consistent with the report of Nweze [12]. It is, however, difficult to clinically distinguish dermatophytosis from other non-mycotic dermatoses and quite often, different dermatophyte species produce similar lesions which are difficult to differentiate through clinical examination [17]. Therefore, identification by direct microscopic examination and *in vitro* culture is required for appropriate diagnosis.

The presence of non-pigmented, septate hyphae in skin scales and ectothrix spores on hair surfaces observed by direct examination in this study agrees with the report of Silveira-Gomes *et al*. [25] who studied 494 human skin scrapings by direct examination and concluded that the presence of arthroconidia in skin scales is diagnostic of dermatophyte involvement.

Although it is a highly efficient screening technique, direct examination is limited in its specificity and sensitivity as false-negative had been reported in 10-15% of samples [17]. Nasimuddin *et al*. [26] found that only 34.35% of the 300 skin scrapings processed for mycology were positive by direct examination while 49.0% were culture positive. Furthermore, it is not possible to identify a fungus up to species level by this method [17]. Since prophylaxis and therapy may vary depending on the species causing the infection [27], the need to isolate the pathogen in culture and its identification at species level is imperative.

Gupta *et al*. [28] used macroscopic features such as the colony pigmentation, topography, texture, and rate of growth coupled with microscopic morphology of fungal elements including the size and shape of macro- and micro-conidia, spirals, nodular organs, and pectinate branches for the identification of dermatophyte species. According to Rosen [29], *T. verrucosum* should be considered if a fungal colony is slow-growing and white, with a smooth folded surface. Colonies of *T*. *verrucosum* in this report were slow-growing, white, heaped, smooth, and slightly folded with some submerged growth and yellow reverse pigment. This observation is consistent with the findings of Rosen [29] and Al-ani *et al*. [20]. It is, however, different from the report of Shams-Gahfarokhi *et al*.[21] who described the growth of *T. verrucosum* on selective agar for pathogenic fungi medium as small, button-like, white cream-colored, with suede-like to velvety surface, a raised center, and flat periphery with some submerged growth. This difference could be as a result of variation in the strains studied and media since colonial characteristics may vary depending on the media used [17].

The broad, irregular hyphae with abundant chlamydoconidia in chains (chains of pearls) typical of *T. verrucosum* as observed under the microscope in this study agree with the report of Rippon [30] who found that chlamydospores of *T. verrucosum* have thick walls and occur in chains. Most strains of *T*. *verrucosum* do not produce conidia [29]. However, the microconidia producing strains have been documented by Yuksel and Likit [31].

In this study, the direct examination method had higher number of positive cases (13.0%) than the culture method (11.0%). This is in agreement with the findings of Moreira *et al*. [32] and that of Bhatia and Sharma [16] who found higher rates by direct examination than by culture when they studied skin samples obtained from dermatophytosis affected rabbits and humans, respectively, and concluded that direct examination was a rapid and efficient method for presumptive diagnosis of ringworm infection. However, it is at variance with the findings of Gupta *et al*. [28] who reported sensitivities of 73.3% and 100% for direct examination and culture, respectively. The lower sensitivity of the culture technique in this report may be due to frequent contamination by saprophytic fungi which might have prevented the growth of the pathogens [33]. Moreover, the authors do not know whether the animals which were positive for dermatophytes by direct examination but negative by culture had been treated with antifungal agents before sample collection. Perhaps, some culture-negative specimens contained residual chemotherapeutic agents due to the previous topical antifungal therapy. Many antifungal drugs used for the treatment of ringworm are retained for long period of time within the horny layer of the epidermis and drug residues present in the samples may prevent the growth of the causative dermatophytes [34]. Furthermore, since the KOH technique cannot differentiate between viable and non-viable fungal elements, chances are that some of these samples might contain dead dermatophytes. These non-viable fungi would not grow in culture and may be responsible for the false-negative culture in spite of a positive direct examination [35]. Robert and Pihet [17] had suggested that insufficient material, very short incubation, or non-suitable temperature could result in a false-negative culture.

The significantly higher prevalence rate of cattle ringworm (p<0.05) in Plateau North senatorial district may be due to the colder, near temperate climatic condition when compared with the prevalence rate obtained in Plateau South senatorial district with considerably warmer climate. This is contrary to the generally accepted belief that the disease is more common in environments with warm and humid climatic conditions [7,12]. This may be due to the higher number of crossbred cattle in this area. It is believed that crossbreed animals are less resistant to dermatophyte infection than local breeds of cattle [36]. It may also be for the same reason that we found significantly higher prevalence of the disease (p<0.05) in the Friesian-Bunaji crossbred than the local Bunaji breed of cattle and thus confirming the report of Swai and Sanka [22] who found a higher rate in the crosses than the indigenous Tanzanian short horn zebu. This may be because the local breeds of animals are likely to have more specific immunity resulting from frequent exposure to local dermatophyte strains and hence more resistant to the disease than the crossbreeds. The significantly higher prevalence of dermatophytosis in calves than adult cattle (p<0.05) in this report suggests that young animals are more likely to acquire infection than older animals. This observation agrees with the findings of Al-Ani *et al*. [20]. Sham-Ghahfarokhi *et al*. [21] and Agnetti *et al*. [23] who reported that fungal infections were more common among cattle less than 6 months of age and that the age of animal was a statistically significant risk factor associated with dermatophytosis. This may be in part due to the weak specific and non-specific immunity and high pH of the skin in young animals. Animal susceptibility is determined largely by immunological status, and hence, young animals may be more susceptible since immunity increases with age [37]. Furthermore, adult animals have more subcutaneous adipose tissues. The breakdown of fat into fatty acid and glycerol lowers the skin pH and makes the adult animal less susceptible to fungal infection [38].

In the present study, there was no significant difference in occurrence of dermatophytosis between male and female cattle (p>0.05). This agrees with the report of Agnetti *et al*. [23] who studied several factors for their roles in bovine dermatophytosis and concluded that age was the only significant risk factor in animals.

The significantly higher prevalence rate was obtained for cattle that were confined (p<0.05) when compared with unconfined animals. This is in agreement with the findings of Ala-ani *et al*. [20] and that of Shams-Ghahfarokhi *et al*. [21] who reported that housing animals in close proximity to each other for long periods in the presence of infected debris were responsible for the high incidence of the disease in winter. This may be because animals in close confinement have restricted movements. The chances of direct contact with one another are higher, especially during the cold season when animals huddle together to keep warm and, consequently, increase transmission [39]. The significantly higher prevalence of the disease was obtained during the rainy season (p<0.05) when compared with the dry season and, therefore, agreeing with the report by Shams-Ghahfarokhi *et al*. [21], Sudan *et al*. [39], and Bhatia and Sharma [16]. This may be attributable to the high humidity resulting from heavy rainfall which favors multiplication of dermatophytes, thereby predisposing animals to infection [39].

The occurrence of ringworm lesions more commonly on the head region in calves agrees with the report of Pandey and Cabaret [40] who studied the distribution of ringworm lesions in cattle naturally infected with *T. verrucosum*. They observed that periocular lesions were more characteristic of young animals, while bulls had more lesions on the dewlap and intermaxillary space. Much earlier reports also showed similar patterns in the distribution of ringworm lesions on the body of cattle. Gentles and O’Sullivan [41] examined 77 infected animals and found lesions on the head (79%), neck (53%), shoulders (15.5%), back (23.5%), lumbar region (15.5%), and tail (9%). According to Ford [42], the neck, head, trunk, and limbs were infected in decreasing order and the preferential sites on the head were around the eyes followed by the ears, cheeks, and face and muzzle. The reason for the occurrence of more lesions around the eyes and face in young animals is not well understood. However, the habit of licking and grooming by calves could predispose these parts of the animal to infection. Furthermore, in sucking calves, these parts of the body are likely to be subject to constant wetting by mammary secretions, especially during suckling. Maceration resulting from continuous wetness could predispose to fungal infection [22]. Apart from the report by Ford [42] and that of Gentles and O’Sullivan [41] to the best of the authors’ knowledge, there are no recent reports in literature regarding the distribution of dermatophytosis lesions on the body of cattle.

## Conclusion

*T. verrucosum* and *T. mentagrophytes* were isolated and identified from skin lesions of cattle with *T. verrucosum* occurring more frequently (54.2%) than *T. mentagrophytes* (45.8%). The head region was the preferred site of dermatophyte infection in young animals. Age, breed, management practice, and season were found to be significant risk factors associated with dermatophytosis of cattle in Plateau State, Nigeria.

## Authors’ Contributions

JSD designed the study, collected, and analyzed the samples. HMK, JKPK, and CNK supervised the project. All authors were involved in data analysis and writing of the manuscript. All authors have read and approved the final manuscript.
